# Climate-driven context-dependent structure of population cycles

**DOI:** 10.1098/rsos.240047

**Published:** 2024-08-28

**Authors:** Noelle I. Samia, Osnat Stramer, Takashi Saitoh, Nils Chr. Stenseth

**Affiliations:** ^1^ Department of Statistics and Data Science, Northwestern University, 2006 Sheridan Road, Evanston, IL 60208, USA; ^2^ Department of Statistics and Actuarial Science, University of Iowa, 241 Schaeffer Hall, Iowa City, IA 52242, USA; ^3^ Field Science Center, Hokkaido University, North 11, West 10, Sapporo 060-0811, Japan; ^4^ Centre for Ecological and Evolutionary Synthesis (CEES), University of Oslo, PO Box 1066, Blindern, 0316 Oslo, Norway

**Keywords:** panels of autoregressive time series, spatio-temporal mixed-effects statistical analysis, ecological interactions, predator–prey cycle, climate, global climate change

## Abstract

Multiannual population cycles of small mammals are of interest within population biology. We propose an approach for multidimensional autoregressive (AR) time series and analyse monitoring data on grey-sided voles (*Myodes rufocanus*) in Japan to investigate one or possibly multiple multiannual cycles that drive population dynamics. Temperature, through modifying rodent communities, is found to be a key factor shaping population dynamics. Warmer areas are the main habitat for other rodent species resulting in low vole abundance/dominance, as opposed to higher vole dominance in colder areas—a pattern associated with the AR structure and population cycle. Vole populations in simple rodent communities exhibit an AR(2) cycle of 2–3 years. In areas with complex rodent communities, vole dynamics follows an AR(4) process and a combination of two cycles with different lengths. The AR structure varies in relatively small spatial scales, thus widening the scope of AR analyses needed. Historically, vole abundance increased in the late 1970s and decreased from the 1980s, with warm winters shown to be associated with the decline of vole abundance in the AR(4) populations. This significant association between the AR order, population dynamics, temperature and rodent community provides insights into the declining trends observed in rodent populations of the Northern Hemisphere.

## 1. Introduction

Royama [1] has long argued that the combination of several ecological interactions, through the delayed density-dependent structure, may cause a cycle in population dynamics. Previous studies on the population dynamics of voles [2–9] have shown, using an ecological model with a delayed density-dependent process of order 2, that (i) populations in colder areas are cyclic, whereas populations in warmer areas are less cyclic; (ii) cyclic populations exhibit stronger delayed density-dependent structure; and (iii) the interval of the rodent cycle is 3–5 years (for a review, see [10]). In the literature, the variation of population fluctuation patterns has been described by the variation of the relative strength of first- and second-order delayed density-dependent coefficients [1], and each of these orders has been thought to correspond to a specific biological interaction. A prevailing hypothesis about the rodent cycle assumes that first-order autoregressive (AR) processes may be generated by generalist predators and second-order by specialist predators and explains that cyclic populations with stronger second-order density-dependent structures may be driven by specialist predators that are more influential in colder regions based on Fennoscandian populations [3]. However, the density-dependent interactions may be more divergent than considered from boreal ecological systems. Density dependence could come from both bottom-up (plant–herbivore) and top-down (predator–prey) trophic interactions as well as intrinsic population mechanisms (e.g. [11,12]). Lambin *et al*. [13] demonstrate that common vole populations in southwest France are not consistent with the geographical extension of the latitudinal gradient from Fennoscandia, although their patterns of population dynamics show a similarity to those from Fennoscandian cyclic rodent populations, and claim that a widely applicable explanation for population cycles should be developed based on ecological instead of geographical grounds. Strann *et al*. [14] demonstrate that the population dynamics of small mammals can be different over short distances and that surrogate climate variables, such as latitude, should be avoided; hence, stressing the need for a better understanding of the local population dynamics with the aim at disentangling the role of ecological factors and cyclicity. This ‘context dependency’ that the strength of biological interaction varies over space and/or time is often associated with population and community dynamics [15]. Cornulier *et al*. [16] demonstrate that the amplitude of the grass-eating vole cycle dampened since the 1980s throughout Europe and was associated with a reduction in winter population growth. The disappearance of cycles may emerge through primary production changes caused by climate warming [17]. Rain-on-snow emphasizes the density-dependent decline of the East European vole (*Microtus levis*) in the context of bottom-up interaction [18], and Gilg *et al*. [19] predicted that the rising temperature would extend the cycle length and decrease the maximum density of a lemming (*Dicrostonyx groenlandicus*) population in association with the top-down interaction. Warming may also alter the seasonality that controls the age at first reproduction and the length of the breeding season of voles, and population dynamics may become less cyclic under longer vegetative periods as suggested by the bank vole (*Myodes glareolus*) [20]. To understand the generality and speciality of the rodent cycle, it is necessary to detail the AR behaviour of rodent populations representing density-dependent interactions and compare them among various ecological systems. The statistical models used so far have not been sufficiently sensitive in detecting the detailed density-dependent structure; thus, populations with complex density-dependent structure that may have more than one multiannual cycle in their population dynamics have not been closely examined.

Our proposed AR spatio-temporal model accounts for heterogeneity across locations and over time, using a reversible jump algorithm under the assumption that the AR order is unknown. Our statistical approach enabled us to classify the panels of time series into distinct categories, such that their dynamic density-dependent structures and cycles revealed (i.e. being AR(2), AR(3) or AR(4)) are more complex than the density-dependent structures of order less than or equal to 2 that are assumed in earlier studies [6,8,21]. This methodology allows us to detect the differences in population dynamic features over short distances and is built with the aim of investigating the ‘complex’ density-dependent cyclical structure that varies according to the ecological context of climate and rodent community.

## 2. Methods

### 2.1. Study area

**Figure 1.**
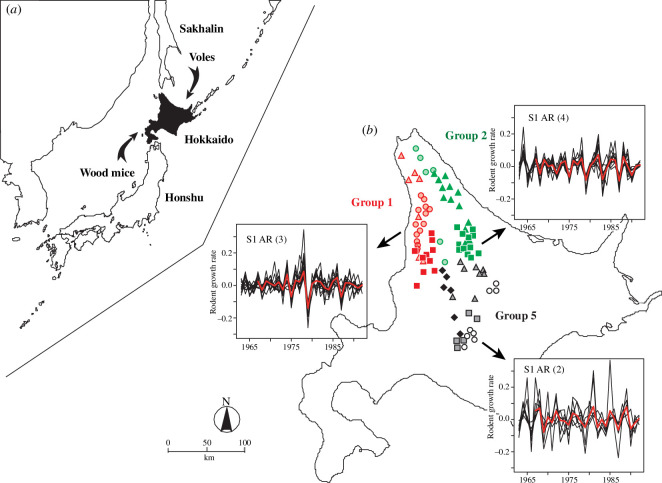
(*a*) Map of the study area: the arrows show immigration routes of rodent species to Hokkaido from Sakhalin or Honshu. (*b*) Group 1 is indicated in red (31 locations), group 2 in green (31 locations) and group 5 in black (27 locations). The subgroup S1 is plotted with circles, subgroup S2 with triangles, subgroup S3 with squares and subgroup S4 with diamonds. AR order 2 is marked with open symbols (i.e. subgroup S1 of group 5), AR order 3 with shaded symbols (i.e. subgroups S1 and S2 of group 1; subgroup S1 of group 2; subgroups S2 and S3 of group 5) and AR order 4 with solid symbols (i.e. subgroup S3 of group 1; subgroups S2 and S3 of group 2; subgroup S4 of group 5). Time-series plots of the observed population growth rate for each AR order are illustrated using subgroup S1 of group 5 (for AR order 2), subgroup S1 of group 1 (for AR order 3), and subgroup S3 of group 2 (for AR order 4). The red curve in each plot represents the fitted time series where the data points $Y_{t-i},i=1,\ldots ,p$ are set at their observed time-point *t* values averaged across all locations in the respective subgroup, the AR coefficients are set at their mean values $\mu_{i},i=1,\ldots ,p$ and the common random effect over time $\eta_{t}$ is set at its estimated mean value for each time point t.

Hokkaido is the northernmost island (41°24′−45°31′ N, 139°50′−145°49′ E) in Japan and covers 78 073 km^2^ (figure 1). The data analysed in this study were obtained from the northern part of the island. The climate on the west coast is warmer than that on the inland and east coast. The winters are snowy throughout the study area. The natural forest in the study area is classified as the ‘pan mixed forest’ with conifers and broad-leaved trees, which is regarded as a transition between the temperate and the subarctic zones [22]. The dominant tree genera are *Abies*, *Acer*, *Betula*, *Picea* and *Quercus*.

The target species is the grey-sided vole, *Myodes rufocanus* (formerly: *Clethrionomys rufocanus* [23]), which is a small-sized rodent (27−50 g). It is widely distributed between Fennoscandia and East Asia [24]. Two species of Japanese wood mice were also common in the study area. The large Japanese wood mouse, *Apodemus speciosus*, is endemic to Japan and is the largest of the three species analysed here (20−60 g for body mass). *Apodemus argenteus* is another endemic Japanese wood mouse, weighing 10−20 g. The grey-sided vole originates from the northern fauna of the Eurasian continent, which consists of species adapting to cooler climates and is considered to have migrated into Hokkaido through Sakhalin [25] (figure 1*a*). *Apodemus* species in Hokkaido originate from the southern fauna, adapting to warmer climates, and are considered to have migrated into Hokkaido through Honshu [25] (figure 1*a*).

*Microtus* species seem to be superior competitors for grey-sided voles on the Eurasian continent [26,27], but they are absent in Hokkaido. Competition between *M. rufocanus* and *Apodemus* has been considered minor because of their different food niches; *M. rufocanus* is folivorous, whereas *Apodemus* is granivorous [28]. For example, *Apodemus speciosus* generally exhibits a peak density after acorn masting, whereas no significant effect of acorn abundance has been detected on the grey-sided voles [29]. However, the detailed interspecific interactions remain unclear. Interactions with other rare species of *Apodemus* mice (*A. peninsulae*) may be negligible. Congeneric interactions with *Myodes rex* and *M. rutilus* are thought to occur [28,30,31], but they are not common in the study area. Shrews (*Sorex unguiculatus* or *S. caecutiens*) are occasionally found in the study area.

Three mustelid species are important predators of small rodents in Hokkaido: *Mustela nivalis* and the two introduced species (*M. itatsi* and *Neogale vison*). *Martes zibellina* and *Mustela erminea* are certainly present in the study area, but there is little information on their predation. The red fox (*Vulpes vulpes*), two owl species (*Strix uralensis* and *Asio otus*) and four snake species (*Elaphe climacophora*, *Elaphe quadrivirgata*, *Euprepiophis conspicillata* and *Gloydius blomhoffii*) are also listed as predators [24]. Among these, *M. nivalis* and *V. vulpes* are considered to be the main predators of voles [32]. A rich alternative prey community (hares, grouses and frogs) also characterizes Hokkaido [33]. Although many parasites are known to infect *M. rufocanus* in Hokkaido [34], their effects on vole population dynamics have not been investigated.

### 2.2. Rodent data

The Forestry Agency of the Japanese Government has surveyed rodent abundance for management purposes because grey-sided voles sometimes destroy large areas of tree plantations [24]. The field survey was carried out by the individual Ranger Office which is a basic unit of forest management. Based on the standard protocol, 50 snap traps were set at 10 m intervals on 0.5 ha (50 × 100 m^2^) grids for three nights in autumn (September/October), and the number of captures was recorded for grey-sided voles and wood mice. Eighty-nine Ranger Offices, each of which had several survey grids, provided a complete series for 31 years (1962−1992).

The trapping efforts reached 1 278 050 trap-nights in 89 locations for 31 years, and 63 437 grey-sided voles and 54 766 wood mice were caught in total. Although the number of traps and survey days varied in this period, the capture records were adjusted to ones expected from the standard protocol; see [32] for details on trapping methods and data arrangement. Therefore, we define the standardized captures for three days on 0.5 ha as density. The mean density of grey-sided voles was 7.6 including all locations and years. The highest was 69 in W-Enbetsu in 1978, and zero was recorded 233 times in 77 locations and in up to 9 years in each location. The highest mean density of grey-sided voles for each location was 18.9 in Teshio, and the lowest mean density (3.7) was recorded in Towari. The dominancy of grey-sided voles in the rodent community is represented by the percentage of grey-sided voles in the total captures and compared between the populations with different AR orders. The vole data were grouped to three geographic groups: the western population in group 1 consisting of 31 locations, the eastern population in group 2 consisting of 31 locations and the southern and mountainous population in group 5 consisting of 27 locations (map in figure 1; see also [5]).

### 2.3. Climate data

**Figure 2.**
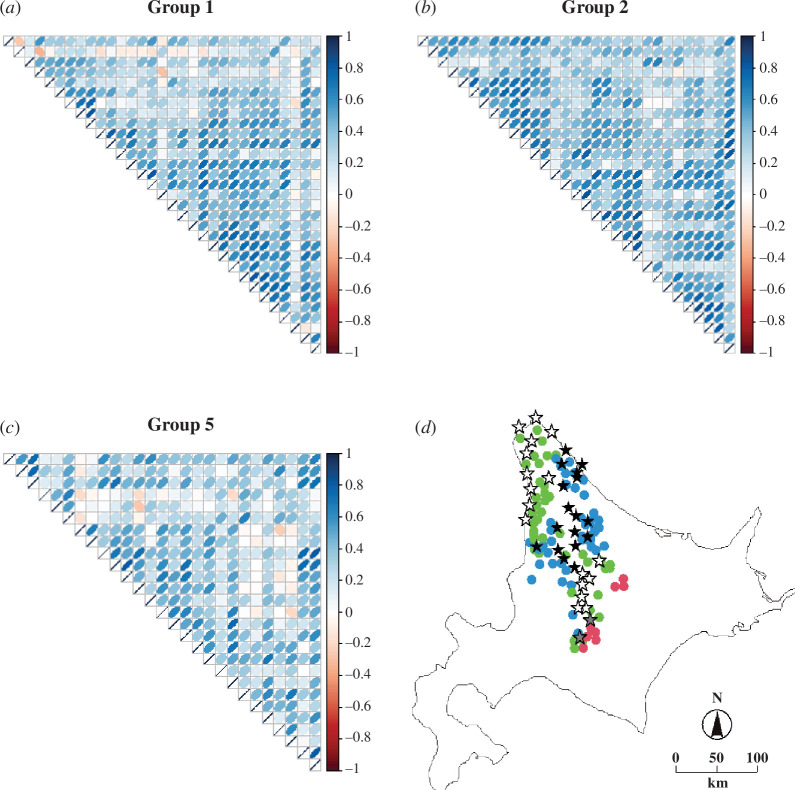
(*a–c*) Correlation matrix heatmap of the correlation between the time series of locations in group 1 (*a*), group 2 (*b*) and group 5 (*c*). The degree of pairwise Pearson’s correlation is displayed by the colour gradient. (*d*) A map of Hokkaido indicating the locations of 89 studied populations and 36 meteorological stations in the study area. The AR order of each population is illustrated by colour: eight populations with the AR order 2 in red, 40 populations with the AR order 3 in green and 41 populations with the AR order 4 in blue. The 36 meteorological stations were grouped into three according to the area in which a particular AR order of the populations dominated: two stations (grey stars) for the populations with the AR order 2, 17 stations (white stars) for the populations with the AR order 3 and 17 stations (black stars) for the populations with the AR order 4.

**Figure 3.**
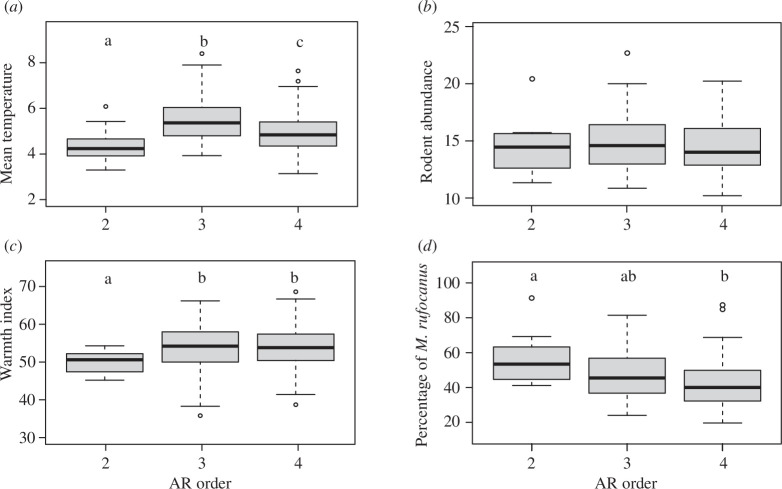
Boxplots of the annual mean temperature (*a*) and the warmth index (*b*) of the three studied areas grouped based on the population AR orders, and those of the number of caught rodents per 150 trap-nights (*c*) and the percentage of the grey-sided voles caught out of the total number of caught rodents in percentage (*d*), based on the population AR orders. Different letters indicate a significant difference between paired AR order groups. No significant difference is found in (*c*).

The meteorological data of 36 stations in figure 2*d* are available on the website of the Japan Meteorological Agency (https://www.data.jma.go.jp/obd/stats/etrn/index.php, last accessed on 12 December 2023). The monthly mean temperatures for 10 years (1980−1989) are analysed. The ‘mean temperature’ in figure 3*a* is the average of monthly mean temperatures for each year. The warmth index in figure 3*b* is an integrated temperature index proposed by Kira [35] for plant growth. Plants require a monthly mean temperature of 5°C or higher for growth in general, and the period of higher temperatures is considered an active season for plant growth. The warmth index is obtained by adding the values of the monthly mean temperature subtracted by 5°C for months with a mean temperature of 5°C or higher [36]. These data were used to characterize the study areas. Six meteorological stations in the study area provide monthly temperatures covering the entire study period (1962−1992). For these meteorological stations, yearly winter temperatures are obtained by averaging the monthly mean temperatures from January to March and yearly summer temperatures by averaging the monthly mean temperatures from April to October. These data were used to analyse the effects of temperatures on vole density in each year of the study period.

### 2.4. Clustering

We analyse the study region by identifying, in each group of locations (groups 1, 2 and 5), distinct homogeneous clusters of sites to which each time series of population growth rate belongs. We calculate the degree of dissimilarity, d, between a pair of time-series datasets based on Pearson’s correlation coefficient r, where d=1-r, if r is statistically significantly different from 0; otherwise, d=1. None of the calculated Pearson’s correlation coefficients is significantly different from 0 and negative; see figure 2*a–c* for correlation matrix heatmaps built using the corrplot package in R [37,38]. The clusters of locations are constructed using the dissimilarity matrix whose components are given by d and the agglomerative hierarchical Ward’s method aiming at finding compact spherical clusters [39]. This is performed using the function hclust in the statistical software R [37]. Preserving a moderate granularity level of clustering in each group, the number of clusters in each of the three dendrograms of groups 1, 2 and 5 is chosen by cutting each dendrogram at a common height equal to the maximum of three heights determined in each dendrogram as follows: the latter dendrogram height is set at the level at which the line with the largest separation between the two main clusters (i.e. the longest vertical line in the dendrogram that does not intersect the merging point) ends just prior to its merging point. For convenience, we choose the heights as one-decimal numbers. For each ensuing cluster or subgroup of locations, we fit, to the population growth rate, the AR mixed-effects model (see §2.5) that accounts for spatio-temporal variations across sites, and we estimate its unknown AR order.

### 2.5. The statistical autoregressive model

Let $V_{k,t}$ denote the total number of grey-sided voles caught and $T_{k,t}$ the corresponding trapping effort in year t and location k. Then, the number of voles standardized by the trapping effort in year t and site k is given by $Z_{k,t}=V_{k,t}/T_{k,t}.$ The yearly population growth rate of voles measures the yearly per cent change in rates and is defined as $Y_{k,t}=\frac{\left(1+Z_{k,t}\right)-(1+Z_{k,t-1})}{1+Z_{k,t-1}}$, where 1 is added because $Z_{k,t-1}$ can be 0 in the denominator; note that $\ln\left(1+Z_{k,t}\right)-\ln\left(1+Z_{k,t-1}\right)$ is approximately equal to $Y_{k,t}$ by Taylor series expansion. We model the yearly growth rate in each subgroup of locations, by introducing and developing the following multidimensional autoregressive time series of dimension *m*, also known as a panel of m autoregressive time series, whose AR order is *p*:


(2.1) $$Y_{kt}=\theta_{0k}+\sum_{j=1}^{p}‍\theta_{jk}Y_{k,t-j}+\eta_{t}+e_{kt},$$

for location k=1,…,m and year $t=1,\ldots ,n_{k}.$ The error terms $e_{k1},e_{k2},\ldots ,e_{k,n_{k}}$ are assumed to be independent and normally distributed with mean 0 and variance $\tau_{k}^{-1}$, where $log(\tau_{k}^{-1})=log(\tau^{-1})+Z$ and Z is $N(0,\sigma_{\tau }^{2}).$ The vector of common effects over time, denoted by $\eta ={\left(\eta_{1},\ldots ,\eta_{n_{\eta }}\right)}^{T}$, with the superscript T denoting the transpose of the vector and where $n_{\eta }={max}_{k=1,\ldots ,m}n_{k}$, is multivariate normal with mean 0 and a variance–covariance matrix $\sigma_{\eta }^{2}I_{n_{\eta }\times n_{\eta }}$, with $I_{n_{\eta }\times n_{\eta }}$ denoting the $n_{\eta }\times n_{\eta }$ identity matrix. We assume that $\left\{e_{kt}\right\}$ and $\left\{\eta_{t}\right\}$ are independent of each other. Let $\theta_{k}=(\theta_{0k},\theta_{1k},\ldots ,\theta_{pk}{)}^{T}$ be a (p+1)-dimensional random vector such that


(2.2) $$\theta_{k}=\mu +\varepsilon_{k},$$

for k=1,…,m, where $\mu ={\left(\mu_{0},\mu_{1},\ldots ,\mu_{p}\right)}^{T}$ is a (p+1)-dimensional vector of fixed effects, and the (p+1)-dimensional vector of random effects $\varepsilon_{k}={\left(\varepsilon_{0k},\varepsilon_{1k},\ldots ,\varepsilon_{pk}\right)}^{T}$ has a multivariate normal distribution with mean 0 and a variance–covariance matrix $\Sigma =\xi I_{\left(p+1)\times (p+1\right)}$. We assume that $\left\{\varepsilon_{k}\right\}$ are independent of each other and of $\left\{e_{kt}\right\}$ and $\left\{\eta_{t}\right\}$.

Hjellvik & Tjøstheim [40] model a panel of m autoregressive time-series data where the autoregressive coefficients and the error variances in model (2.1) above are assumed to be fixed and identical across different locations. We generalize their model to allow for heterogeneity across locations and our results can be easily extended to the case where the common effects over time $\eta_{t}$ are not necessarily independent and identically distributed. We address this inference problem within the Bayesian framework assuming furthermore that the correct AR order p is unknown. We develop reversible jump Markov chain Monte Carlo (MCMC) algorithms. Our approach is based on a version [41] of the reversible jump algorithm of Green [42]. The estimation of the parameters is obtained using a series of Metropolis within Gibbs steps, since while it is possible to derive the full conditional posterior distribution for most of the parameters, it is not possible to derive it for all parameters.

#### 2.5.1. Notations

Let $Y=\left(Y_{1},\ldots ,Y_{m}\right)$, where $Y_{k}={\left(Y_{k,1},Y_{k,2},\ldots ,Y_{k,n_{k}}\right)}^{T}$ is an $n_{k}$-dimensional vector of responses, and $Y_{k,t}$ is defined in equation (2.1) above. Let $\theta =\left(\theta_{1},\ldots ,\theta_{m}\right)$, $\theta_{-k}=\left(\theta_{1},\ldots ,\theta_{k-1},\theta_{k+1},\ldots ,\theta_{m}\right)$ and $\tau_{-k}=\left(\tau_{1},\ldots ,\tau_{k-1},\tau_{k+1},\ldots ,\tau_{m}\right).$ Let $\Phi_{p}$ be the vector of all parameters; i.e. $\Phi_{p}=\left(\;\;\mu ,\theta_{k},\tau ,\tau_{k},\sigma_{\tau }^{2},\sigma_{\eta }^{2},\eta ,\xi ;k=1,\ldots ,m\right).$ Denote by $\Phi^{-}$ the portion of $\Phi_{p}$ that is independent of p; i.e. $\Phi^{-}=\left(\tau ,\tau_{k},\sigma_{\tau }^{2},\sigma_{\eta }^{2},\eta ,\xi ;k=1,\ldots ,m\right).$ Denote by $\Phi_{p}^{+}$ the portion of $\Phi_{p}$ that depends on p; i.e. $\Phi_{p}^{+}=\left(\mu ,\theta_{k};k=1,\ldots ,m\right).$ For $t=1,\ldots ,n_{k}$ and i=1,…,p+1, let $X_{k}$ be an $n_{k}\times (p+1)$ design matrix corresponding to the autoregressive coefficients (including the intercept) given by


$$X_{k}(t,i)=\left\{ \begin{array}{ll} 1 & \;if\;\;i=1\;\\ Y_{k,t-\left(i-1\right)} & \;otherwise.\; \end{array}\right.$$

For $i=1,\ldots ,n_{k}$ and $j=1,\ldots ,n_{\eta }$, let $Z_{k}$ be an $n_{k}\times n_{\eta }$ design matrix ($n_{\eta }=max_{k=1,\ldots ,m}n_{k}$) corresponding to the common effect over time η given by


$$Z_{k}(i,j)=\left\{ \begin{array}{ll} 1 & \;if\;\;i=j\leq n_{k}\;\\ 0 & \;otherwise.\; \end{array}\right.$$

Hence, the model defined in equation (2.1) can now be written as $Y_{k}=X_{k}\theta_{k}+Z_{k}\eta +e_{k}.$

#### 2.5.2. Priors

The following priors are chosen; see electronic supplementary material for a further discussion.

— $\tau^{-1}$ is inverse gamma (a,scale=b).
— $\sigma_{\tau }^{2}$ is inverse gamma $\left(a_{\sigma },{scale=b}_{\sigma }\right)$.
— $\sigma_{\eta }^{2}$ is inverse gamma $\left(a_{\eta },{scale=b}_{\eta }\right)$.
— ξ is inverse gamma $\left(a_{\xi },{scale=b}_{\xi }\right)$.
— μ is multivariate normal (u,V).
— $p(p)\propto \frac{1}{(p+1)!}$ for $p=1,2,\ldots ,p_{max}.$

#### 2.5.3. Reversible jump Markov chain Monte Carlo

To jump between models with different AR orders p, we apply the reversible jump method in Godsill [41] to the model defined in equation (2.1). This method is applicable when $p(\Phi_{p}^{+}|\Phi^{-};Y)$ is available analytically. Suppose that the current state is $\left(p,\Phi_{p}\right)$. We update p as follows:

— Propose a new order $p^{\prime }$ with probability $q\left(p\to p^{\prime }\right)=\frac{exp\left(-\lambda \;|p^{\prime }-p|\right)}{\sum_{p^{\prime }=1}^{p_{max}}‍exp\left(-\lambda \;|p^{\prime }-p|\right)}$ for $p^{\prime }=1,2,\ldots ,p_{max}$ and λ>0.
— Using a similar approach to that in Troughton & Godsill [43], the probability to accept this move is α=min(1,r), where $r=\frac{p\left(p^{\prime }|\Phi^{-},Y\right)}{p\left(p|\Phi^{-},Y\right)}\frac{q\left(p^{\prime }\to p\right)}{q\left(p\to p^{\prime }\right)}=\frac{p\left(p^{\prime }|\Phi^{-},Y\right)}{p\left(p|\Phi^{-},Y\right)},$ and $p(p|\Phi^{-},Y)$ is defined in equations (A 3) and (A 4) in the electronic supplementary material.

#### 2.5.4. Updating $\Phi^{-}$

We use the Gibbs sampler algorithm to update the parameters in $\Phi^{-}$ based on the following conditional distributions:

— $\sigma_{\tau }^{2}|\tau ,\tau_{k},\sigma_{\eta }^{2},\eta ,\xi ,\mu ,\theta_{k},Y$ ~ inverse gamma with shape parameter $a_{\sigma }+\frac{m}{2}$ and scale parameter $b_{\sigma }+\frac{1}{2}\sum_{k=1}^{m}‍[log(1/\tau_{k})-log(1/\tau ){]}^{2}.$
— $\sigma_{\eta }^{2}|\tau ,\tau_{k},\sigma_{\tau }^{2},\eta ,\xi ,\mu ,\theta_{k},Y$ ~ inverse gamma with shape parameter $a_{\eta }+\frac{n_{\eta }}{2}$ and scale parameter $b_{\eta }+\frac{1}{2}\eta^{T}\eta .$
— $\eta |\tau_{k},\tau ,\sigma_{\tau }^{2},\sigma_{\eta }^{2},\mu ,\xi ,\theta_{k},Y$ ~ multivariate normal with mean $=[\frac{1}{\sigma_{\eta }^{2}}I_{n_{\eta }\times n_{\eta }}+\sum_{k=1}^{m}‍\tau_{k}Z_{k}^{T}Z_{k}{]}^{-1}[\sum_{k=1}^{m}‍\tau_{k}Z_{k}^{T}(Y_{k}-X_{k}\theta_{k})]$ and variance $=[\frac{1}{\sigma_{\eta }^{2}}I_{n_{\eta }\times n_{\eta }}+\sum_{k=1}^{m}‍\tau_{k}Z_{k}^{T}Z_{k}{]}^{-1}.$
— $\xi |\tau_{k},\tau ,\sigma_{\tau }^{2},\sigma_{\eta }^{2},\eta ,\mu ,\theta_{k},Y$ ~ inverse gamma with shape parameter $a_{\xi }+\frac{m(p+1)}{2}$ and scale parameter $b_{\xi }+\frac{1}{2}\sum_{k=1}^{m}‍(\theta_{k}-\mu {)}^{T}(\theta_{k}-\mu ).$

We use Metropolis within Gibbs sampler to update the remaining parameters in $\Phi^{-}$ based on the following conditional distributions:

— $\tau |\tau_{k},\sigma_{\tau }^{2},\sigma_{\eta }^{2},\eta ,\xi ,\mu ,\theta_{k},Y$ ~ density of gamma with parameters a+1 and b×(1/τ)× density of $N\left(\frac{1}{m}\sum_{k=1}^{m}‍log(1/\tau_{k}),\frac{\sigma_{\tau }^{2}}{m}\right)$ evaluated at log(1/τ).
— $\tau_{k}|\tau_{-k},\tau ,\sigma_{\tau }^{2},\sigma_{\eta }^{2},\eta ,\xi ,\mu ,\theta_{k},Y$ **~** density of gamma with parameters $\frac{n_{k}}{2}+1$ and $\left(\frac{1}{2}\{(Y_{k}-X_{k}\theta_{k}-Z_{k}\eta {)}^{T}(Y_{k}-X_{k}\theta_{k}-Z_{k}\eta )\}\right)\times$$\left(1/\tau_{k}\right)\times$ density of $N\left(log(1/\tau ),\sigma_{\tau }^{2}\right)$ evaluated at $log(1/\tau_{k}).$

To simulate from the conditional distribution of τ (or $\tau_{k}$), we use the Metropolis–Hastings algorithm with proposal $log\left(1/\tau \right)\sim N\left(\frac{1}{m}\sum_{k=1}^{m}‍log(1/\tau_{k}),\frac{\sigma_{\tau }^{2}}{m}\right)$ [or $log\left(1/\tau_{k}\right)\sim N\left(log(1/\tau ),\sigma_{\tau }^{2}\right)$ for $\tau_{k}$].

#### 2.5.5. Updating $\Phi_{p}^{+}$

We use the Gibbs sampler algorithm to update $\Phi_{p}^{+}=(\mu ,\theta )$ based on $p(\Phi_{p}^{+}|\Phi^{-},Y)=p(\mu |\Phi^{-},Y)\times p(\theta |\mu ,\Phi^{-},Y)$, as follows:

— $\mu |\Phi^{-},Y$ ~ multivariate normal with mean =${\left\{V^{-1}+\frac{m}{\xi }I_{\left(p+1\right)\times \left(p+1\right)}-\frac{1}{\xi^{2}}\sum_{k=1}^{m}‍{\left(\frac{1}{\xi }I_{\left(p+1\right)\times \left(p+1\right)}+\tau_{k}X_{k}^{T}X_{k}\right)}^{-1}\right\}}^{-1}$

$\times \left\{V^{-1}u+\frac{1}{\xi }\sum_{k=1}^{m}‍\tau_{k}{\left(\frac{1}{\xi }I_{\left(p+1\right)\times \left(p+1\right)}+\tau_{k}X_{k}^{T}X_{k}\right)}^{-1}X_{k}^{T}\left(Y_{k}-Z_{k}\eta \right)\right\}$ and variance

$={\left\{V^{-1}+\frac{m}{\xi }I_{\left(p+1)\times (p+1\right)}-\frac{1}{\xi^{2}}\sum_{k=1}^{m}‍{\left(\frac{1}{\xi }I_{\left(p+1)\times (p+1\right)}+\tau_{k}X_{k}^{T}X_{k}\right)}^{-1}\right\}}^{-1}.$

— $\theta_{k}|\theta_{-k},\mu ,\Phi^{-},Y$ ~ multivariate normal with mean $=[\frac{1}{\xi }I_{\left(p+1)\times (p+1\right)}+\tau_{k}X_{k}^{T}X_{k}{]}^{-1}[\frac{1}{\xi }\mu +\tau_{k}X_{k}^{T}(Y_{k}-Z_{k}\eta )],$ and variance $=[\frac{1}{\xi }I_{\left(p+1)\times (p+1\right)}+\tau_{k}X_{k}^{T}X_{k}{]}^{-1}.$

#### 2.5.6. Simulation study

A simulation study is conducted to demonstrate the efficacy of our approach; results are shown in the electronic supplementary material.

### 2.6. Effects of climate on voles

**Figure 4.**
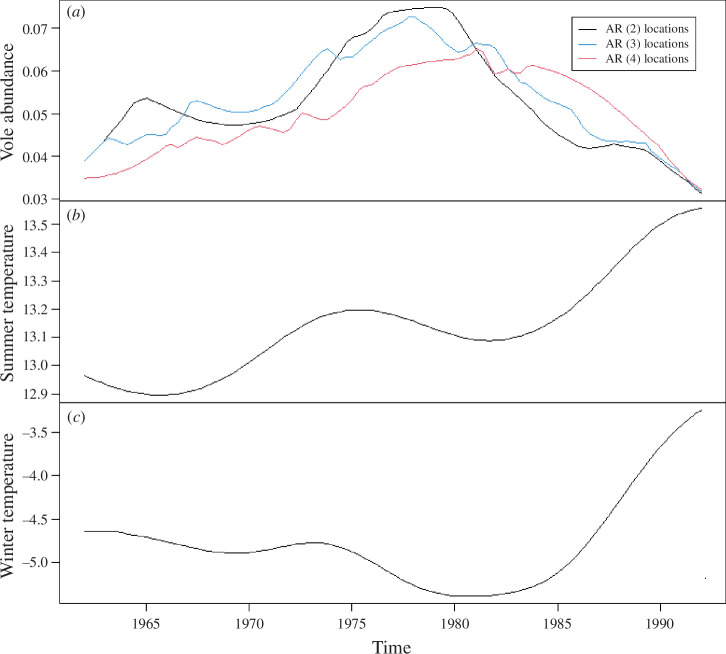
Time-series plots of the kernel-smoothed 50% quantiles of the vole abundance in AR(2), AR(3) and AR(4) locations indicated in black, blue and red curves, respectively (*a*), of the kernel-smoothed 50% quantiles of yearly summer temperature for six meteorological stations (*b*) and of the kernel-smoothed 50% quantiles of yearly winter temperature for six meteorological stations (*c*).

To obtain insight into the biological interactions, we analyse the relationship between the AR order and temperature (the annual mean temperature and the warmth index) [36], and the relationship between the AR order and the abundance and dominance of voles, using the analysis of variance. In addition, we use a quantile regression modelling approach to further study the effects of the temperature on the vole abundance in each of the AR(2), AR(3) and AR(4) locations [44]; see electronic supplementary material. A median quantile regression of the kernel-smoothed 50% quantiles of yearly vole abundance of the AR(2)–AR(4) locations on the kernel-smoothed 50% quantiles of yearly winter and summer temperatures is shown in figure 4.

## 3. Results

We detect substantial differences in the population dynamics of the voles in our data across sites in each geographical group (groups 1, 2 and 5) by identifying distinct homogeneous clusters of locations to which each time series of population growth rate belongs (figure 1*b*). We fit, to the population growth rate in each subgroup, the newly introduced AR mixed-effects model that accounts for spatio-temporal variations across panels of time-series data, and we estimate its corresponding AR order, resulting in an AR order that differs across the subgroups of sites (electronic supplementary material, table S1; figure 1*b*). We find that group 1 is divided into two subgroups of AR order 3 and one subgroup of AR order 4; group 2 is divided into one subgroup of order 3 and two subgroups of order 4; group 5 is divided into one subgroup of order 2, two subgroups of order 3 and one subgroup of order 4. Electronic supplementary material, table S1, summarizes, for each subgroup of locations, the MCMC output of the parameters in the grey-sided vole model, i.e. the posterior mean, the posterior standard deviation and the test statistic of Geweke’s convergence diagnostics. Based on the model fitting and diagnostics (see figure 1*b*; electronic supplementary material), our statistical model fits the data closely; a comparative study and criteria used for checking the adequacy of the fitted model are described in the electronic supplementary material.

The AR(2) populations (in group 5) are located inland, which is significantly colder than the coastal areas where the AR(3) and AR(4) populations dominated (figures 1 and 3). We analyse the effects of the AR order on the annual mean temperatures from 36 meteorological stations for ten years using a mixed-effects model with random meteorological stations and observation years. The AR order is found to be significantly associated with the variation in annual mean temperatures. The Tukey multiple comparison test based on the simple one-way model indicates that the mean temperature significantly differs in every paired comparison. The mean temperature is the highest in the area where the populations with AR(3) dominate and the lowest where AR(2) dominates. The mean temperature is intermediate in the area where the populations with AR(4) dominate; see also boxplots in figure 3.

Populations of AR orders 3 and 4 are located in areas with higher plant growth; see boxplots of warmth index in figure 3*b* (median warmth index is 50.6, 54.2 and 53.8 for vole populations of AR orders 2, 3 and 4, respectively). The warmth index analysed in the same manner as the mean temperature shows the significant effects of the AR order. The Tukey multiple comparison test based on the simple one-way model indicates that the warmth index is significantly lower in the area where the populations with the AR order 2 dominate than the other two areas; see also boxplots in figure 3*b*.

The boxplots (figure 3*c,d*) of rodent abundance and proportion of voles caught out of the total number of caught rodents show a significantly high dominance of voles in the AR(2) populations against the AR(4) populations (median vole rate is 58, 51 and 46% for AR orders 2, 3 and 4, respectively). The analysis of variance based on the simple one-way model shows a significant difference in the vole rate between AR orders 2 and 4. Other comparisons are not statistically significant, and rodent abundances are similar between those categories (median rodent abundances are 14.5, 14.6 and 14.0 for AR orders 2, 3 and 4, respectively).

**Figure 5.**
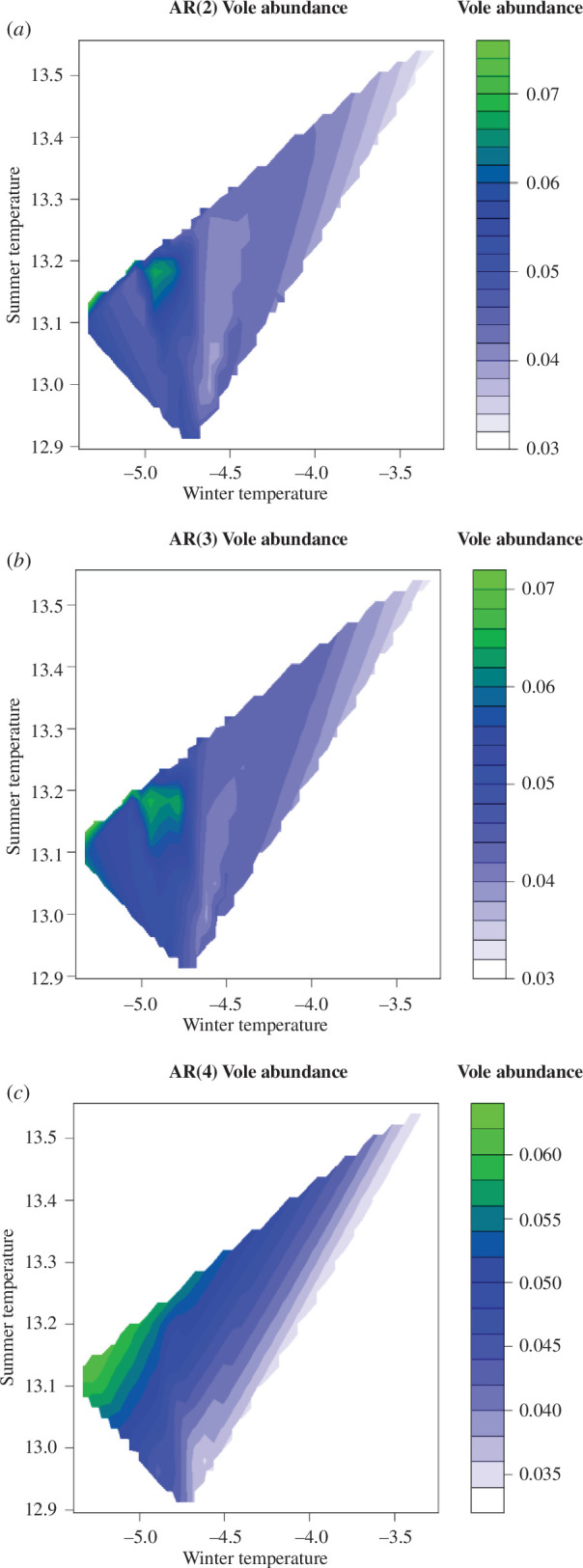
Contour plots displaying the effects of the summer temperature and the winter temperature on the vole abundance in the AR(2) locations (*a*), the AR(3) locations (*b*) and the AR(4) locations (*c*). The vole abundance and the summer and winter temperatures are represented by the kernel-smoothed 50% quantiles of yearly values.

To summarize the observed pattern, AR orders from AR(2) to AR(4) correspond to temperature (from cooler to warmer) and to the vole dominancy (from dominant to less dominant). Figure 4*f* shows that the vole abundance increased in the late 1970s and decreased from about 1980, while the winter temperature fell in the late 1970s and rose from the late 1980s. The summer temperature gradually increased with some fluctuations. The fitted median quantile models in the electronic supplementary material and the time-series plots in figure 4 show that the winter temperature has a significant negative effect and the summer temperature has a significant positive effect on the vole abundance. The same is true when we use a similar median quantile regression approach on the kernel-smoothed 50% quantiles of vole abundance for the AR(3) and AR(4) locations shown in figure 4. We find that pseudo-*R*^2^ temperatures show the highest impacts on vole abundance in the AR(4) group (pseudo-*R*^2^ = 0.837), followed by the AR(3) group whose pseudo-*R*^2^ is 0.550, and it is lowest for the AR(2) group with a value of 0.320. The winter temperature has a negative effect under the constant summer temperature, while the effect of summer temperature is positive under the constant winter temperature. These relationships are confirmed in the contour plots (figure 5). In addition, the contour plots illustrate the differences in temperature effects between the three AR groups. The summer temperature zones with high vole abundance are consistent at 13.1−13.2°C through AR groups. The winter temperature zones with high vole abundance vary between the three AR groups (figure 5). The AR(4) populations show the highest abundance in the coldest period below −5°C (figure 5*c*), whereas the highest abundances of the AR(2) and AR(3) populations are observed in the periods with −5°C and −4.8°C (figure 5*a*). The AR(2) populations inhabit the coldest areas, where the interaction with the wood mouse is minor. The convex relationship between vole abundance and winter temperature observed in the AR(2) populations (figure 5*a*) may be attributed to the low performance of the regression model for the AR(2) populations (pseudo-*R*^2^ = 0.320). The AR(3) populations that inhabit the intermediate temperature areas show the intermediate features between the AR(2) and AR(4) populations in the relationship of the vole abundance to temperatures (figure 5*b*; electronic supplementary material).

The approximate length of cycles originating from an AR(2) population (i.e. subgroup S1 of group 5) is 3.3 years (electronic supplementary material, table S1). The AR(3) population shows a cyclic behaviour of an average length of 3.76 years and an exponentially decaying feature of the population growth rate. However, the AR(4) populations exhibit a combination of two cycles with average lengths of 2.52 and 4.89 years. For a description of how to estimate the length of the periodic cycles and how to detect the exponentially decaying feature, see electronic supplementary material.

## 4. Discussion

By fitting the AR mixed-effects model where the AR order is assumed to be unknown and is estimated by our modelling approach and using the data, we find a higher order density-dependence structure (AR orders 3 and 4; electronic supplementary material, table S1; figures 1 and 2*d*). Our developed statistical model demonstrates the importance of higher order density-dependence structure that highlights the cyclicity in the data and quantifies the length of each cycle observed. Below, we discuss how the differences in population cycles, as identified in our study as resulting from AR(2), AR(3) or AR(4) processes, may correspond to three different rodent assemblages in different areas. The fact that the AR(2) populations are located in cooler areas where wood mouse species are scarce (figure 3) suggests that the dynamics of the AR(2) populations is formed by simpler ecological interaction. The AR(2) populations could access their favourite resources without disturbance, and predators may concentrate on grey-sided voles more intently in this simple rodent assemblage. In contrast, the AR(3) and AR(4) populations are distributed in warmer areas with suitable habitats for mice species (figure 3).

The winter temperature may be attributed to explaining the differences in the model performance between the three AR groups of populations. The wood mouse species are of southern origin, while the Hokkaido vole is of northern origin. Therefore, the severe winter temperatures may be harsher to the wood mouse species than the Hokkaido vole. Low winter temperatures may suppress the activity of wood mice; the large Japanese wood mouse exhibits daily torpor under artificial winter conditions [45]. In contrast, lower winter temperatures are favourable for the Hokkaido vole, even though these temperatures are not best for them, because the interference from the wood mice may be reduced. Therefore, higher vole abundances are observed in the periods with lower winter temperatures in the areas for AR(4) populations where mouse species are dominant (figure 5*c*). This straightforward relationship may be attributed to the high performance of the quantile regression model for vole abundance to temperature (pseudo-*R*^2^ = 0.837).

The rodent survey was carried out in natural forests, which were favourable habitats to studied rodents and held similar abundances of rodents regardless of the AR orders (figure 3*c*). A higher abundance of wood mice inhabiting the AR(4) areas results in a low dominance of voles and, hence, some interspecific interactions are suggested in rodent assemblages. Until recent years, the population dynamics of rodents have been discussed within the context of AR(2) consisting of density effects from one and two years before (e.g. [10]). The life span of small rodents is generally short. Grey-sided voles hardly survive two winters and can directly carry physiological and/or psychological effects of density only from the previous to the current year. The maternal effects could transfer older density effects; nutritionally rich mothers who grew up under a favourable density without severe resource competition may produce highly reproductive young ones, whereas individuals born from females who grew up under high densities may be poor for survival and reproduction. These correspond to one- and two-year lag effects of density. Similarly, generalist and specialist predators have also been considered possible agents of one- and two-year lag effects of density. The trophic interactions between voles and food plants (e.g. bilberries in northern Eurasia) could also exhibit a similar function although a detailed mechanism has not been identified yet.

This study demonstrates that higher orders of AR were prevailing in the studied vole populations and appeals to widen the scope of density effects on rodent populations. The noticeable effects from the third and fourth AR orders indicate that the density information from three and four years before should be kept as delayed-density effects. Such delayed-density effects with three- or four-year lag cannot be expected to be kept within vole populations because of their short life span, and external mechanisms keeping vole density effects should be considered. The decreasing trend in the vole dominancy from AR(2) to AR(4) in figure 3*d* suggests that grey-sided vole populations with higher AR orders may suffer more strongly from wood mice. Wood mice are granivorous, differing from the folivorous food niche of the grey-sided vole in Hokkaido, and their populations change following seed masting. The large Japanese wood mouse generally exhibits a peak density after acorn masting of *Quercus crispula*, whereas no significant effect of acorn abundance was detected on the grey-sided vole and the other sympatric wood mouse (*A. argenteus*) population [29]. The different responses to acorn masting between the three sympatric rodent species can be explained by the variation in tannin tolerance [46]. Only the large Japanese wood mouse can mitigate the toxic effects of tannins contained in acorns and positively respond to acorn masting. Therefore, acorn abundance may indirectly affect vole density through large Japanese wood mice in the AR(4) areas where grey-sided voles are less dominant. Acorn masting has been observed at two- to three-year intervals in Hokkaido [46]; high abundance rarely occurs in two successive years, while low abundance sometimes continues for successive years. When taking acorn masting as a background cycle, we could explain the combination of the first and second cycles observed in AR(4) populations (electronic supplementary material, table S1). We understand that this discussion is speculative. However, we know little about the time delays of density-dependent factors (food resources, competitors and predators) involved in the rodent cycle, and such discussion may contribute to encouraging researchers to add more factors than previously considered into a list of candidate factors.

In earlier studies, density-dependent structure has been discussed in relation with latitude [2,3], the combined index of latitude and longitude [5] and altitude [9]. Although latitude, longitude and altitude could be a proxy of weather conditions, weather conditions may vary more complexly than the variation represented by those variables. We substantially extend the report by Strann *et al*. [14] showing that the features of the cycle (i.e. the AR structure) are different over short distances (20−30 km) and that the use of surrogate climatic variables should be reconsidered. Low temperature is associated with a low proportion of wood mouse, and the ecological interactions modified by the temperature result in a density-dependent structure of vole populations, as determined by our statistical approach.

Our temporal analyses show that the changes in winter temperatures roughly matched the changes in vole abundance across the entire study area (figure 4). The winter temperatures fell in the late 1970s and rose from the late 1980s, while vole abundance increased in the late 1970s and decreased from about 1980. The amplitude of the vole population fluctuations in our area of study dampened since the 1980s. Decreasing trends of abundance and damped oscillation have been observed in rodent populations of the Northern Hemisphere [16,47]. It is a mystery why rodent populations are losing cyclicity in some places but not others. Ehrich *et al*. [47] found that lemming populations co-occurring with one or several species of voles outside Fennoscandia were declining, but no decreasing trends were evidenced in general. Cyclicity is consistent over long periods (50 years) in the Kola Peninsula, Russia [48] and Yukon, Canada [49]. We indicate that the AR structure could vary in a relatively small region (northern Hokkaido) and warm winters are associated with the decline of vole abundance. Therefore, local weather conditions may be attributed to the spatial variation of vole dynamics through altering rodent assemblages. Ehrich *et al*. [47] suggest the effects of community structure including predators on the spatial variation of population dynamics observed in lemming populations (see also [50]). Spatio-temporal analyses of the association between population dynamics and winter temperature are promising to untangle the mystery of the spatial and temporal variation of cyclicity.

## Data Availability

The monitoring rodent data and code are made available in the electronic supplementary material [51].
